# Atherogenic index of plasma is a novel and better biomarker associated with obesity: a population-based cross-sectional study in China

**DOI:** 10.1186/s12944-018-0686-8

**Published:** 2018-03-05

**Authors:** Xiaowei Zhu, Lugang Yu, Hui Zhou, Qinhua Ma, Xiaohua Zhou, Ting Lei, Jiarong Hu, Wenxin Xu, Nengjun Yi, Shufeng Lei

**Affiliations:** 1 Center for Genetic Epidemiology and Genomics, School of Public Health, Medical College of Soochow University, Suzhou, Jiangsu 215123 China; 2 Jiangsu Key Laboratory of Preventive and Translational Medicine for Geriatric Diseases, School of Public Health, Medical College of Soochow University, Suzhou, Jiangsu 215123 China; 3 Center for Disease Control and Prevention of Suzhou Industry Park, Suzhou, Jiangsu 215021 China; 4 No. 3 People’s Hospital of Xiang Cheng District, Suzhou, Jiangsu 215134 China; 5 Department of Biostatistics, University of Alabama at Birmingham, Birmingham, AL 35294 USA

**Keywords:** Obesity, Atherogenic index of plasma, Blood lipid components

## Abstract

**Background:**

Atherogenic index of plasma (AIP) has been reported to be associated with cardiovascular diseases. However no study has yet systematically evaluated the association between AIP and obesity and its advantage in obesity prediction compared with conventional lipid components.

**Methods:**

A total of 6465 participants aged over 30 years were included in this study. Blood lipid components including triglyceride (TG), total cholesterol (TC), high-density lipoprotein cholesterol (HDL-C) and low-density lipoprotein cholesterol (LDL-C) were measured, and AIP was calculated as log_10_(TG/HDL-C). Pearson correlation analyses, multivariable logistic analyses and predictive analyses were used to evaluate the association and discrimination ability between AIP, four conventional lipid profiles and obesity.

**Results:**

Subjects in the higher quartiles of AIP all had a significantly increased risk of obesity compared with those in the lowest quartile (*P* for trend< 0.01). AIP showed a stronger association with obesity than the conventional lipid components as the pearson coefficient reached up to 0.372 and the adjusted odds ratio was 5.55. Using AIP rather than HDL-C and TG significantly improved risk prediction for obesity (AUC improvement = 0.011, *P* = 0.011; Continuous net reclassification index = 29.55%, *P* < 0.01; Category net reclassification index = 6.06%; Integrated discrimination improvement = 0.68%, *P* < 0.01).

**Conclusions:**

Higher AIP level was positively and strongly associated with obesity. AIP is a novel and better biomarker associated with obesity. Controlling the AIP level would be more helpful for the prevention of obesity.

## Background

Obesity has become a global problem of public health over the past few decades with its high prevalence (range between 11% and 15%) and medical burden in both developed and developing countries [1]. As generally known, obesity is considered as a medical condition in which excess body fat has been accumulated. The storage room of body fat that adipocytes and adipose tissue also store the greatest amount of body lipids including triglycerides and free cholesterol [2]. Recent years, most studies have reported that blood lipids and obesity are closely related [3–5]. Looking for the optimal blood lipid index to be used as a novel biomarker for obesity will make up for the limitation of body mass index (BMI) acted as a simple anthropometric indicator of obesity. The lipid biomarker as a direct biological target will also be helpful in obesity prevention and therapeutic treatment.

Atherogenic index of plasma (AIP) is a novel index composed of triglycerides and high-density lipoprotein cholesterol [6]. It has been used to quantify blood lipid levels and commonly used as optimal indicator of dyslipidemia and associated diseases (e.g., cardiovascular diseases) [7–9]. However, no published study has yet examined the association between AIP and obesity, and especially the discrimination ability between AIP, conventional lipids and obesity risk. Our study thus aimed to assess the association between AIP, the conventional lipid components and obesity in a large-scale Chinese population and further systematically highlighted the predictive advantage of AIP for obesity compared with the conventional lipid components.

## Methods

### Study participants

The study subjects were recruited via a local health physical examination program conducted in two municipal districts of Suzhou in southeast of China, during June 2013 to December 2013. Participants enrolled in the study met the criteria of age over 30 years and Chinese Han ethnicity. After excluding subjects for lacking blood samples, a total of 6465 subjects were finally included in the analysis. The study was approved by the ethical committee of Soochow University. Subjects agreeing to participate into the present study provided a written informed consent.

### Data collection

Health examination was performed in the morning after the examinees fasted overnight. Anthropometric indices were measured by an eligible physician. Weight was measured in light indoor clothing without shoes, using a calibrated balance beam scale, and height was measured using a calibrated stadiometer. Body mass index (BMI) was calculated as body weight (in kilograms) divided by the square of height (in meters). Obesity was defined as BMI ≥ 28 kg/m^2^ according to the criteria of China Obesity Task Force [10]. Seated blood pressure was measured by mercury sphygmomanometer after 10-min rest in the sitting room.

Peripheral blood was drawn into an EDTA-containing tube and stored into a 4 °C refrigerator, and was subjected to biochemical experiments within 24 h. Glucose oxidase method (Homa, Beijing, China) was used to detect fasting blood glucose (FBG). Blood lipid indexes including triglyceride (TG), total cholesterol (TC), high-density lipoprotein cholesterol (HDL-C) and low-density lipoprotein cholesterol (LDL-C) were measured by Hitachi 7180 autoanalyzer (Hitachi, Tokyo, Japan). Atherogenic index of plasma (AIP) was calculated as logarithmic transformation of the ratio of TG to HDL-C.

### Statistical analysis

All participants were divided into obesity and non-obesity groups as well as the quartile groups based on their AI*P* values (<− 0.231, − 0.231 to − 0.052, − 0.053 to 0.146, ≥0.147). The baseline variables were compared using the Student’s t-test, one-way ANOVA, or the chi-squared test, as appropriate. The Pearson correlation analyses were used to assess the relationship between AIP, four lipid components and BMI in total participants.

Univariate and multivariate logistic regression analyses were conducted to evaluate the association between AIP, four lipid components and obesity. Total participants were further categorized into four groups according to the quartiles of AIP. The odds ratio (OR) along with its 95% confidence interval (95%CI) of obesity were estimated for higher three categories of AIP with the lowest one as a reference. A linear trend in the ORs of obesity was performed by entering AIP quartiles as continuous parameters in the model. Area under the curve (AUC) of receiver operating characteristic (ROC), net reclassification index (NRI) and integrated discrimination improvement (IDI) were calculated to compare the predictive value between AIP and conventional lipid profiles including TG and HDL for predicting obesity. All statistical tests were two-tailed and were considered significant for p less than 0.05. Statistical analyses were performed using SAS statistical software (version 9.4, SAS Institute, Cary, NC, USA) and R software (version 3.0, the R Foundation for Statistical Computing, Vienna, Austria).

## Results

Among 6465 participants included in the study, 503 of them (7.78%) were obesity. As shown in Table 1, participants with obesity were more likely to have higher systolic blood pressure (SBP), diastolic blood pressure (DBP), FBG, TG, TC, LDL-C and AIP as well as a lower level of HDL-C. In Table 2, the participants with higher AIP tended to be younger and have higher BMI, DBP, FBG, TG, TC, LDL-C as well as lower HDL-C.

**Table 1:** Baseline Characteristics between Obesity and Non-obesity Group among Total Population

	Obesity	Non-obesity	*P* value
Number	503	5962	
Men, n(%)	225(44.7%)	2797(46.9%)	0.35
Age, years	60.21 ± 12.15	61.2 ± 12.64	0.09
Systolic blood pressure, mmHg	141.89 ± 19.83	134.99 ± 21.49	< 0.01
Diastolic blood pressure, mmHg	86.67 ± 10.65	82.74 ± 11.5	< 0.01
Fasting blood glucose, mmol/L	6.33 ± 1.54	5.94 ± 1.21	< 0.01
Triglyceride, mmol/L	1.97 ± 1.18	1.48 ± 1.07	< 0.01
Total cholesterol, mmol/L	5.18 ± 0.94	4.95 ± 0.98	< 0.01
HDL cholesterol, mmol/L	1.35 ± 0.34	1.45 ± 0.38	< 0.01
LDL cholesterol, mmol/L	2.98 ± 0.74	2.77 ± 0.73	< 0.01
Atherogenic index of plasma	0.13 ± 0.26	−0.04 ± 0.28	< 0.01

Note: Variables are expressed as the mean ± standard deviation. HDL cholesterol, high-density lipoprotein cholesterol; LDL cholesterol, low-density lipoprotein cholesterol

**Table 2:** Baseline Characteristics according to Atherogenic Index of Plasma Quartile among Total Population

	Quartile 1 < − 0.231	Quartile 2 (− 0.231)-(− 0.052)	Quartile 3 (− 0.053)-(0.146)	Quartile 4 ≥ 0.147	*P* value
Number	1614	1619	1618	1614	
Men, n(%)	807(50%)	727(44.9%)	712(44%)	776(48.1%)	0.23
Age, years	63.6 ± 12.26	62.16 ± 12.7	60.53 ± 12.49	58.21 ± 12.33	< 0.01
Body mass index, kg/m^2^	21.8 ± 2.93	22.95 ± 2.97	23.92 ± 2.99	25.01 ± 2.94	< 0.01
Systolic blood pressure, mmHg	136.27 ± 21.52	135.68 ± 21.95	135.06 ± 21.55	135.11 ± 20.74	0.33
Diastolic blood pressure, mmHg	82.3 ± 11.93	82.69 ± 11.53	83 ± 11.21	84.18 ± 11.18	< 0.01
Fasting blood glucose, mmol/L	5.8 ± 1.06	5.91 ± 1.19	5.95 ± 1.13	6.2 ± 1.51	< 0.01
Triglyceride, mmol/L	0.76 ± 0.18	1.09 ± 0.21	1.5 ± 0.33	2.72 ± 1.54	< 0.01
Total cholesterol, mmol/L	4.87 ± 0.88	4.92 ± 0.95	4.99 ± 1	5.08 ± 1.05	< 0.01
HDL cholesterol, mmol/L	1.76 ± 0.38	1.51 ± 0.28	1.35 ± 0.26	1.15 ± 0.3	< 0.01
LDL cholesterol, mmol/L	2.5 ± 0.6	2.75 ± 0.67	2.93 ± 0.73	2.97 ± 0.83	< 0.01

Note: Variables are expressed as the mean ± standard deviation. HDL cholesterol, high-density lipoprotein cholesterol; LDL cholesterol, low-density lipoprotein cholesterol

**Fig. 1:**
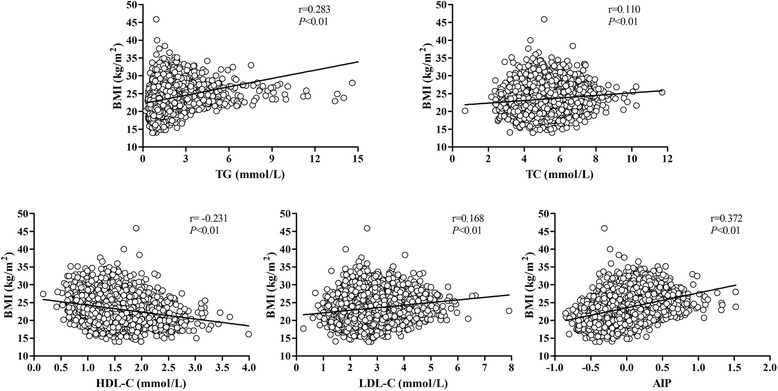
Scatterplot showing significant correlations of four lipid components and AIP to BMI

Figure 1 showed the significant linear correlations between AIP, four lipid profiles and BMI. TG, TC, LDL-C and AIP positively correlated to BMI while HDL-C inversely correlated to it. Among them, AIP showed a strongest association with BMI as the coefficient reached up to 0.372 (*P* < 0.01).

In Table 3, univariate analyses showed the significant associations between AIP, four lipid profiles and obesity (*P* < 0.01). After adjustment for age, gender, SBP, DBP and FBG, the associations remained significant (*P* < 0.01). Both in these two models, AIP had a highest OR of obesity compared with the other lipid components. After further adjustment for other lipid components, AIP remained to have a strongest association with obesity. In addition, participants in higher AIP quartiles all had a significantly increased risk of obesity compared with the reference group with the ORs of 2.573, 3.765 and 6.205, respectively (*P* for trend < 0.01).

**Table 3:** Odds Ratios (95% Confidence Interval) of Obesity according to Lipid Components

	unadjusted	*P* value	multivariable adjusted*	*P* value	multivariable adjusted**	*P* value
TG	1.295(1.217–1.378)	< 0.01	1.229(1.154–1.308)	< 0.01	1.123(1.029–1.226)	< 0.01
TC	1.253(1.147–1.369)	< 0.01	1.196(1.088–1.315)	< 0.01	1.207(0.963–1.512)	0.103
HDL-C	0.45(0.348–0.584)	< 0.01	0.375(0.283–0.496)	< 0.01	0.354(0.233–0.539)	< 0.01
LDL-C AIP	1.445(1.287–1.622)	< 0.01	1.391(1.237–1.566)	< 0.01	1.113(0.873–1.420)	0.386
	6.691(4.956–9.034)	< 0.01	5.829(4.272–7.952)	< 0.01	5.550(4.03–7.644)	< 0.01
AIP Quartiles						
<−0.231	1.000(Reference)		1.000(Reference)		1.000(Reference)	
(−0.231)-(− 0.052)	2.732(1.85–4.036)	< 0.01	2.672(1.806–3.954)	< 0.01	2.573(1.735–3.813)	< 0.01
(− 0.053)-(0.146)	4.152(2.859–6.029)	< 0.01	4.014(2.758–5.842)	< 0.01	3.765(2.575–5.506)	< 0.01
≥0.147	7.358(5.142–10.53)	< 0.01	6.689(4.652–9.618)	< 0.01	6.205(4.293–8.968)	< 0.01
*P* for trend	< 0.01		< 0.01		< 0.01	

Note. TG, TC, HDL-C, LDL-C and AIP indicate triglyceride, total cholesterol, high-density lipoprotein cholesterol, low-density lipoprotein cholesterol and atherogenic index of plasma. * Multivariable model included age, gender, systolic blood pressure (SBP), diastolic blood pressure (DBP), fasting blood glucose (FBG) and any one lipid component.** Multivariable model included two models. One model included TG, HDL-C, TC, LDL-C, age, gender, SBP, DBP and FBG. In this model, the ORs (95%CIs) of TG, TC, HDL-C and LDL-C were calculated. The other model included AIP, TC, LDL-C, age, gender, SBP, DBP and FBG. In this model, the ORs (95%CIs) of AIP and its quartiles were calculated

**Fig. 2:**
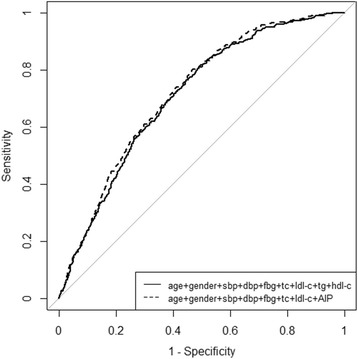
Discriminatory powers for obesity between AIP and conventional lipid components including TG and HDL-C levels by receiver operating characteristic (ROC) curves

As shown in Fig. 2 and Table 4, compared with the model including TG, HDL-C, age, gender, SBP, DBP, FBG, TC and LDL-C, the model including AIP, age, gender, SBP, DBP, FBG, TC and LDL-C significantly improved the accuracy of risk prediction for obesity. After replacing TG and HDL-C with AIP, the AUC for the model improved by 0.011 (AUC changed from 0.706 to 0.717; *P* = 0.011). The continuous and category NRIs were 29.55% (*P* < 0.01) and 6.06% (*P* < 0.01), and the IDI was 0.68% (*P* < 0.01).

**Table 4:** Comparison of Discrimination Performance for Obesity between AIP and Conventional Lipid Components including TG and HDL-C Levels

	NRI (Continuous), %	NRI (Category), %^*^	IDI, %
Conventional model adding TG and HDL-C	1.00(Reference)	1.00(Reference)	1.00(Reference)
Conventional model adding AIP	29.55(20.57–38.52)	6.06(3.06–9.07)	0.68(0.5–0.86)
*P* value	< 0.01	< 0.01	< 0.01

Notes: TG, HDL-C and AIP indicate triglyceride, high-density lipoprotein cholesterol and atherogenic index of plasma. The conventional model included age, gender, systolic blood pressure, diastolic blood pressure, fasting blood glucose, total cholesterol and low-density lipoprotein cholesterol

*Patients were divided into three categories for risk classification: < 5%, 5%–15%, and > 15%. NRI, net reclassification improvement; IDI, integrated discrimination index

## Discussion

In this large-sample cross-sectional survey, our findings indicated that subjects with higher AIP levels tended to have a higher risk of obesity. We also for the first time detected that integrating the two indies (TG and HDL-C) to generate the composite one of AIP is more likely to increase the predictive sensibility for obesity. AIP can be considered as a novel and better biomarker for obesity.

AIP, calculated as log_10_(TG/HDL-C), was initially constructed as a biomarker of plasma atherosclerosis [6] and has been proved to be significantly correlated with other important atherosclerosis indexes such as LDL-C size and small-dense LDL-C [11, 12]. To date, quite a few studies have detected an association between AIP and atherosclerosis-related conditions. For example, Abdullah et al. and Cure et al. demonstrated that AIP was significantly and positively correlated with carotid intima-media thicknesses (r = 0.304, *P* < 0.001; r = 0.261, *P* < 0.001) [13, 14]. A large-sample case-control study in China also indicated that AIP was significantly associated with coronary artery disease with an adjusted OR (95%CI) of 1.66 (1.367–2.016) [9]. Although a previous study reported a higher prevalence of obesity associated with AIP elevating, it did not adjust other confounding factors and compare the magnitude of the association between AIP, conventional lipid profiles and obesity [15].

The conventional lipid components are recommended to be used in atherosclerotic cardiovascular diseases [16, 17]. Several lipoprotein ratios have been established and widely used in recent years. The balance constructed between atherogenic and protective lipoproteins makes the lipoprotein ratios have greater cardiovascular risk than conventional lipid components used alone [18]. Among those ratios, AIP has been reported to be a better biomarker for coronary artery disease [9] and type 2 diabetes [19] than the conventional lipids. In our study, the Pearson analyses, logistic analyses and especially predictive analyses including AUC, NRI and IDI all indicated that AIP was a useful and superior biomarker for obesity. Enhancing the control of AIP rather than conventional lipids may better prevent obesity prevalence in the total population.

Our study has a few advantages that deserve mentioning. At first, this is a large-scale survey research in an adult population of China, and it proves AIP has the superior predictive power than conventional lipid profiles for obesity for the first time. All information collection and laboratory assay were conducted with strict quality controls. In addition, the findings of a strongest association between AIP and obesity as well as the predictive power of AIP superior to the conventional lipids were stable and reliable because of a high statistically power. The integration of the two indies (TG and HDL-C) to generate a composite one of AIP for obesity could avoid the inconsistent assessment of different lipid components and simplify the prediction task in practical application. Furthermore, as obesity is generally recognized as the important risk factor of many diseases, AIP could be considered as a novel and applicable biomarker for the related diseases incidence and prognosis. Future drug discovery for obesity and related diseases could be conducted with AIP as a treatment target.

There were also some limitations that should be mentioned. First, the feature of cross-sectional study contributed to the difficulty in building the definite causal relationship between blood lipid profiles and obesity. However, it is reasonable for AIP to be an independent risk factor for obesity because of the biological association between blood lipid profiles and obesity. Second, the data of other confounders including diet, medical and drug history, tobacco and alcohol consumption as well as physical activity were not included in this analysis because of the information default. The possibility that these variables confounded the true association between AIP and obesity cannot be completely eliminated. We will include these relevant variables in the further researches and combine them with AIP together in obesity prediction. Third, the levels of lipid profiles in our study were determined with a single measurement. Therefore, some exposure misclassification risk is inevitable but is probably low, as previous work demonstrated that lipid components are stable over time [20].

## Conclusions

Higher AIP level was positively and strongly associated with obesity. AIP is a novel and better biomarker associated with obesity. It can be used in clinical practice as a direct biological target to evaluate the effect of hypolipidemic drugs on the prevention and treatment of obesity, which may facilitate clinicians to adjust the medication plan in order to reduce the adverse reactions caused by long-term use of lipid-lowering drugs.

## Abbreviations

AIP: Atherogenic index of plasma;
AUC: Area under the curve
BMI: Body mass index
DBP: Diastolic blood pressure
FBG: Fasting blood glucose
HDL-C: High-density lipoprotein cholesterol
IDI: Integrated discrimination improvement
LDL-C: Low-density lipoprotein cholesterol
NRI: Net reclassification index
OR: odds ratio
ROC: Receiver operating characteristic
SBP: Systolic blood pressure
TC: Total cholesterol
TG: Triglyceride
